# Prevalence, burden, and clinical management of migraine in China, Japan, and South Korea: a comprehensive review of the literature

**DOI:** 10.1186/s10194-019-1062-4

**Published:** 2019-12-05

**Authors:** Takao Takeshima, Qi Wan, Yanlei Zhang, Mika Komori, Serina Stretton, Narayan Rajan, Tamas Treuer, Kaname Ueda

**Affiliations:** 1 Department of Neurology, Headache Center, Tominaga Hospital, Osaka, Japan; 2 Department of Neurology, Jiangsu Province Hospital, Nanjing, China; 3 Eli Lilly and Company, Shanghai, China; 4 Medicine Development Unit-Japan, Eli Lilly Japan K.K., 5-1-28, Isogamidori, chuo-ku, Kobe, 651-0086 Japan; 5 ProScribe Envision Pharma Group, Sydney, Australia; 6 Eli Lilly Australia Pty. Ltd., Sydney, Australia; 7 GPORWE International, Lilly Hungaria Kft, Budapest, Hungary

**Keywords:** Burden, China, Cost of illness, Epidemiology, Far East, Hong Kong, Japan, Korea, Medical economics, Migraine disorders, Prevalence, Quality of life, Taiwan

## Abstract

**Background:**

The objective of this review was to determine the unmet needs for migraine in East Asian adults and children.

**Methods:**

We searched MEDLINE and EMBASE (January 1, 1988 to January 14, 2019). Studies reporting the prevalence, humanistic and economic burden, and clinical management of migraine in China (including Hong Kong and Taiwan), Japan, and South Korea were included. Studies conducted before 1988 (before the International Headache Society [IHS] first edition of the International Classification of Headache Disorders) were not included.

**Results:**

We retrieved 1337 publications and 41 met the inclusion criteria (28 from China, 7 from Japan, and 6 from South Korea). The 1-year prevalence of migraine (IHS criteria) among adults ranged from 6.0% to 14.3%. Peak prevalence ranged from 11% to 20% for women and 3% to 8% for men (30- to 49-year-olds). For children, prevalence of migraine increased with age. Information on the economic burden and clinical management of migraine was limited, particularly for children. When reported, migraine was significantly associated with high levels of disability and negative effects on quality of life. Studies suggested low levels of disease awareness/diagnosis within each country. Of individuals with migraine from China, 52.9% to 68.6% had consulted a physician previously, 37.2% to 52.7% diagnosed with headache had not been diagnosed with migraine previously, and 13.5% to 18% had been diagnosed with migraine previously. Of individuals with migraine from Japan, 59.4% to 71.8% had never consulted a physician previously, 1.3% to 7.3% regularly consulted physicians for their headache, and only 11.6% of individuals with migraine were aware that they had migraine. In addition, studies suggested that over-the-counter medication use was high and prescription medication use was low in each country.

**Conclusions:**

This review suggests that there are unmet needs for migraine in terms of sufficient and appropriate diagnosis, and better management and therapies for treatment of migraine in East Asia. The findings are limited by a lack of recent information and significant gaps in the literature. More recent, population-based studies assessing disease burden and clinical management of migraine are needed to confirm unmet needs for migraine across East Asia.

## Introduction

Migraine is a disabling primary headache disorder that places an enormous burden on patients and society [1–7]. The impact of migraine extends beyond the physical pain of a migraine attack and can have substantial effects on multiple aspects of an individual’s life, including day-to-day functioning [7–9]. Findings from the Global Burden of Disease Study found migraine to be the second highest cause of years lost due to disability, interfering significantly with occupational, educational, household, family, and social responsibilities [10], and the second highest contributor to neurological disease burden, after stroke [11].

Most data on migraine burden worldwide are derived from epidemiological surveys conducted mostly in the United States and Europe [1–3, 5, 6, 12–14]. However, disease burden can vary significantly by geography and ethnicity, particularly for chronic conditions such as migraine in which pain is a major contributor to disability [15]. Two reviews on migraine and headache prevalence have been conducted in the Asia-Pacific region, including countries from East Asia, Southeast Asia, and West Asia [16, 17]. These reviews were focused on the prevalence of chronic migraine and chronic daily headache, and on headache disorders across multiple countries, with little information on migraine-related burden.

The objective of this comprehensive literature review of the evidence related to the prevalence, burden, and clinical management of migraine in East Asia (China, Japan, and South Korea) was to determine the unmet needs for migraine in both adults and children. The key outcomes were prevalence, disease burden (humanistic or economic), and clinical management of migraine, including health care utilization and clinical practice patterns.

## Materials and methods

### Data sources and search terms

The following online databases were searched: MEDLINE via Ovid (1988 to January 14, 2019), EMBASE via Ovid (January 1, 1988 to January 14, 2019). Search strategies were developed by an author (SS), adapted for each database, and included keywords (Medical Subject Heading or EMTREE) and free-text terms for the following subjects: Migraine disorders or chronic daily headache, China (including Taiwan and Hong Kong), South Korea, and Japan. Because small numbers of publications were retrieved with these terms, specific terms for disease burden were not included in the electronic search strategy. For each database, electronic searches were limited to studies conducted in humans.

### Eligibility criteria

Publications were included if they reported on participants with migraine from China (including Hong Kong and Taiwan), South Korea, or Japan. There were no restrictions on the definition of migraine, age group included, or language of publication. All studies were to include at least one of the following outcomes of migraine: prevalence, humanistic burden (all reported measures of health-related quality of life [HRQoL], migraine-related disability, and measures of the impact of migraine on aspects of daily living and social activities), economic burden (work-related productivity, direct and indirect medical costs), and clinical management (health care utilization, clinical practice patterns). Randomized and nonrandomized clinical studies, long-term follow-up studies, and prospective and retrospective observational studies were included for all outcomes except prevalence. For studies reporting prevalence, only population-based studies were included.

Publications were excluded for the following reasons: studies conducted or published before 1988 (before the International Headache Society [IHS] first edition of the International Classification of Headache Disorders [ICHD-1]) [18] and before the availability of triptans, which were the first disease-specific medications for acute treatment of migraine); reported on participants with mixed headache types in which data for those with migraine were not reported separately; the race/ethnicity of the study population was not reported; populations of interest were not reported separately in studies on mixed populations, or populations of interest resided in other countries; were preclinical, animal, and other nonclinical experimental studies; were case studies or series, review articles, letters to the editor, consensus papers or guidelines, and congress abstracts; reported on duplicate data; reported on outcomes for fewer than 30 participants; or did not report on the prespecified outcomes. Reference lists from relevant systematic reviews and meta-analyses that were retrieved in the search strategy were screened manually to identify publications not retrieved by the literature search strategies.

### Screening and data extraction

Searches were collated using bibliographic management software. An initial screen of the title and abstract of each publication retrieved from the search strategy was conducted by one author (SS) to remove duplicate publications and identify potential publications that met the criteria for migraine and eligible country. Inclusion of each potential publication was then confirmed after a review of the full text to identify publications reporting one or more of the following eligible outcomes: prevalence of migraine, humanistic burden, economic burden, and clinical management as described above. All authors were consulted in instances when inclusion was uncertain, and authors reviewed and approved the final list of articles identified for inclusion in the review. One author (SS) extracted all data into prespecified data tables. A second independent reviewer (non-author) checked all extracted data, and any disagreements were resolved by consensus. The data extracted included study characteristics, population characteristics, criteria for migraine diagnosis, prevalence of migraine, outcomes measuring or describing migraine burden (humanistic, work-related productivity, indirect and direct medical costs), health care utilization, and clinical practice patterns.

## Results

### Literature search output

**Fig. 1:**
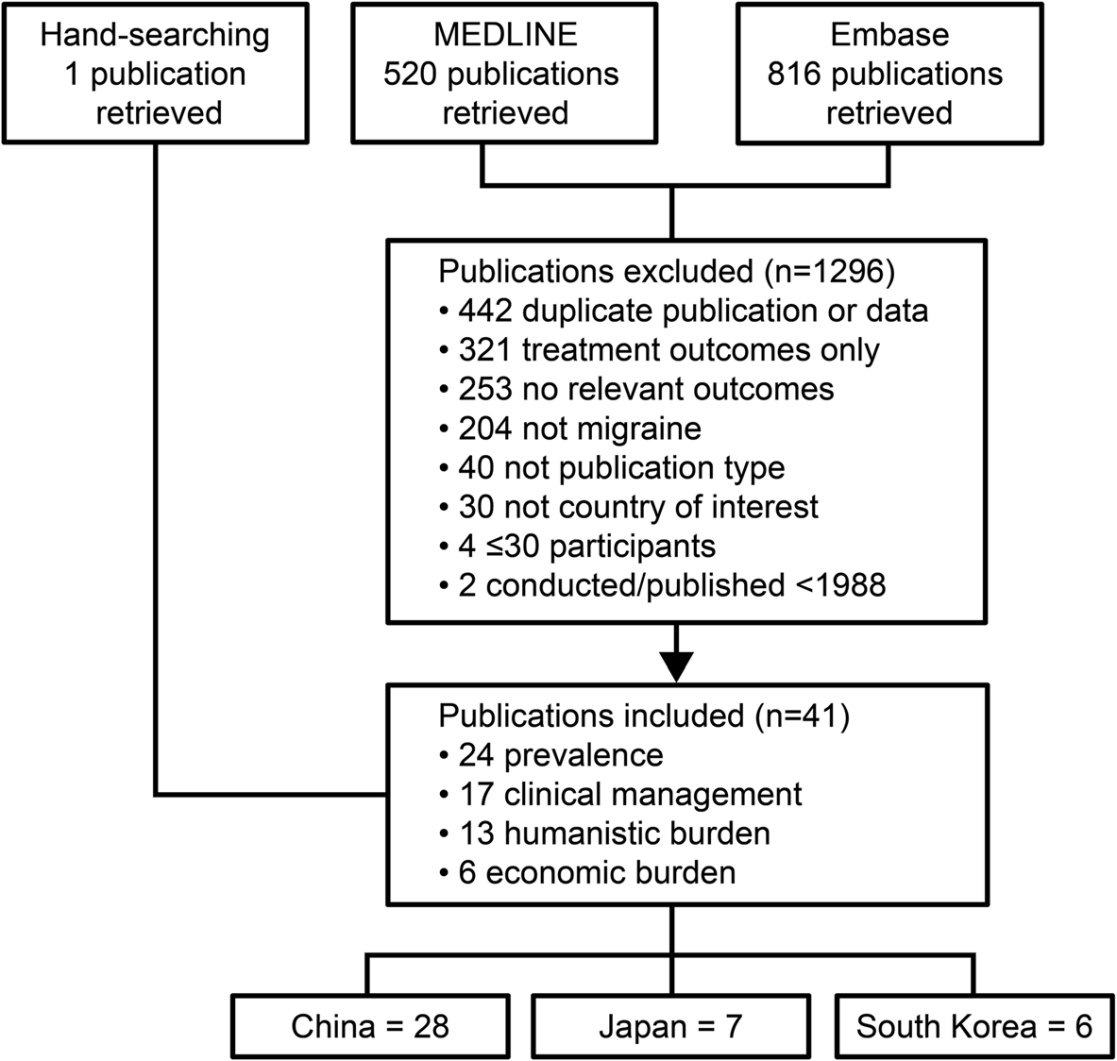
Publication flow Note: publications were excluded for 1 reason but may have met > 1 exclusion criterion; most included publications reported outcomes for ≥2 topics.

A total of 1337 publications were retrieved: 1336 by electronic searching and 1 by hand searching (Fig. 1). The 3 main reasons for exclusion were duplicate publications/publications reporting duplicate data, treatment outcomes only, and no relevant outcomes. A total of 41 publications were identified for inclusion: 28 from China, including China mainland [19–30], Hong Kong [31, 32], and Taiwan [33–46], 7 from Japan [47–53], and 6 from South Korea [54–59]. The included publications were published between 1995 and 2018 and reported on studies conducted between 1993 and 2018. Only 4 studies were conducted within the last 5 years.

### Prevalence of migraine: population-based studies

There were 23 population-based studies (24 publications) reporting the prevalence of migraine from a specified geographic area or community (see Additional file 1); 11 were in adults, 2 in elderly adults (≥60 years), 2 in young adults (university students), and 8 in schoolchildren and/or adolescents.

Most of the population-based studies identified migraine according to IHS [18] criteria (ICHD-1, ICHD-II, or ICHD-IIIβ) (see Additional file 1). Two studies used modified migraine criteria and included attacks of 2- to 4-h duration [43, 51]. Shorter headache durations in subjects who fulfilled all other IHS criteria were used in these studies because almost all subjects took medication for their headache or slept to alleviate headache and, therefore, it was not possible to confirm durations longer than 4 h [43, 51]. One study included migrainous headache not fulfilling ICHD migraine criteria [59], and 3 studies used the Chinese version of the ID Migraine Screener [19, 26, 27] to diagnose migraine.

### Prevalence of migraine in adults

**Fig. 2:**
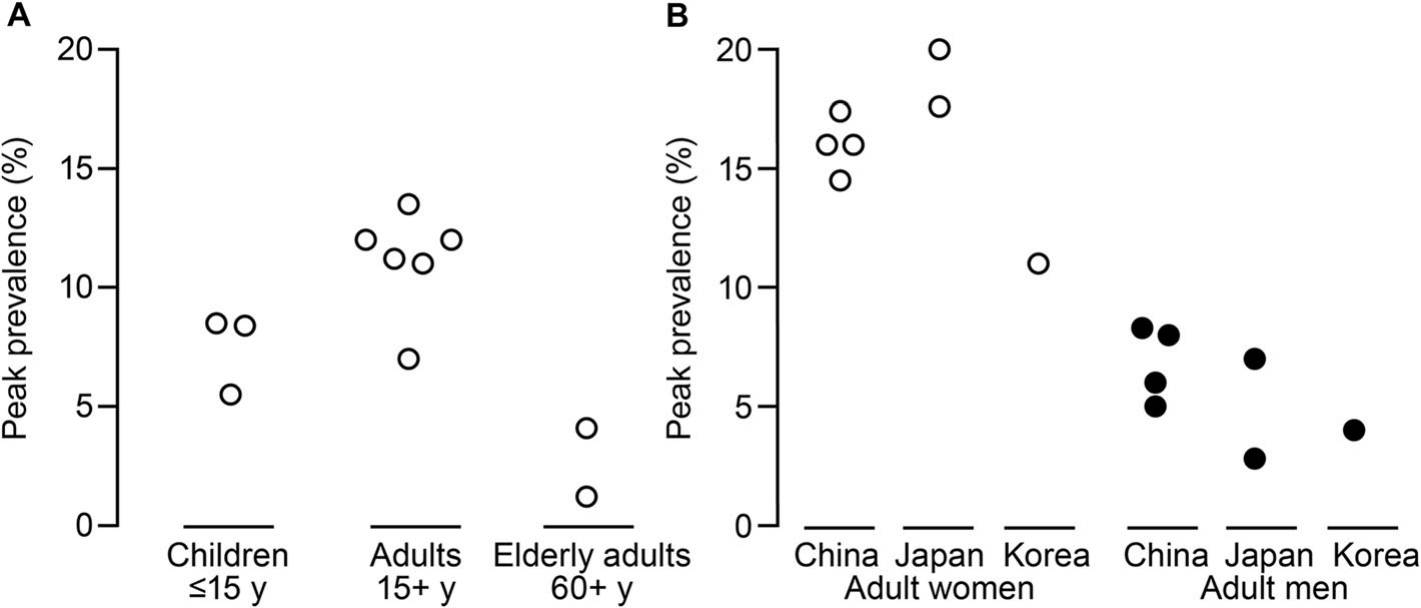
Publications reporting peak prevalence (A) by age groups and (B) by country. Data in (A) were derived as follows: children [31, 36, 40], adults [20, 23, 27, 29, 43, 56], and elderly adults [30, 45]. Note, because of the age groups enrolled in each study, there was overlap in populations among the children, adult, and elderly adult categories. The prevalence of migraine in children aged 16–18 years from Roh et al. 2012 (14.2%) [58] is not included because of the overlap in age with adults. Data in (B) were derived as follows: China [20, 27, 29, 43], Japan [51, 53], and South Korea [56]. Roh et al. 1998 [59] used a non-standard definition of migraine for adults in South Korea and is not reported in panels A or B

Of the studies reporting prevalence of migraine according to IHS criteria, the 1-year prevalence among non-elderly adults ranged from 6.0% in Japan to 14.3% in mainland China (see Additional file 1). Crude estimates of prevalence ranged from 4.7% in Hong Kong to 10.5% in an ethnic minority group in mainland China. Although there was variation in peak prevalence among the studies and between countries, the peak prevalence among non-elderly adults ranged from 7% among 40- to 49-year-olds in South Korea to 13.5% among 30- to 34-year-olds in Taiwan and was highest for women (Fig. 2; see Additional file 1). Across all studies in non-elderly adults, the peak prevalence of migraine according to IHS criteria ranged from 11% to 20% for women and from 2.8% to 8.3% for men, most typically among 30- to 49-year-olds (see Additional file 1). The peak prevalence in elderly adults was 1.2% and 4.1% among 60- to 69-year-olds from China mainland and Taiwan, respectively (see Additional file 1). There appeared to be no change over time (from 1993 to 2013) in the peak prevalence of migraine among adult men or women (data not shown).

### Prevalence of migraine in children

Comparisons of schoolchildren by age or over time suggested that the prevalence of migraine increased with increasing age [36, 58] (see Additional file 1). The prevalence of migraine from a study in Taiwan was 4.8%, 7.1%, and 8.4% for ages 13, 14, and 15 years, respectively [36], and from a study in South Korea was 4.9%, ~ 9%, and 14.2% for age ranges 6 to 12, 13 to 15, and 16 to 18 years, respectively [58]. The prevalence of migraine (ICHD-IIIβ) among junior and senior school students in Japan was 3.5% for 6- to 12-year-olds and 5.0% for 12- to 15-year-olds [48]. When reported, the prevalence of migraine was consistently higher for girls than boys [36, 40, 47, 58]. The peak prevalence of migraine among children up to 15 years of age was lower compared with the prevalence in adults, but higher than in elderly adults (Fig. 2). However, comparisons of peak prevalence among children are difficult because of the different age groups enrolled in each study, which for some studies overlapped with the adult populations (see Additional file 1).

### Humanistic burden of migraine in adults

**Fig. 3:**
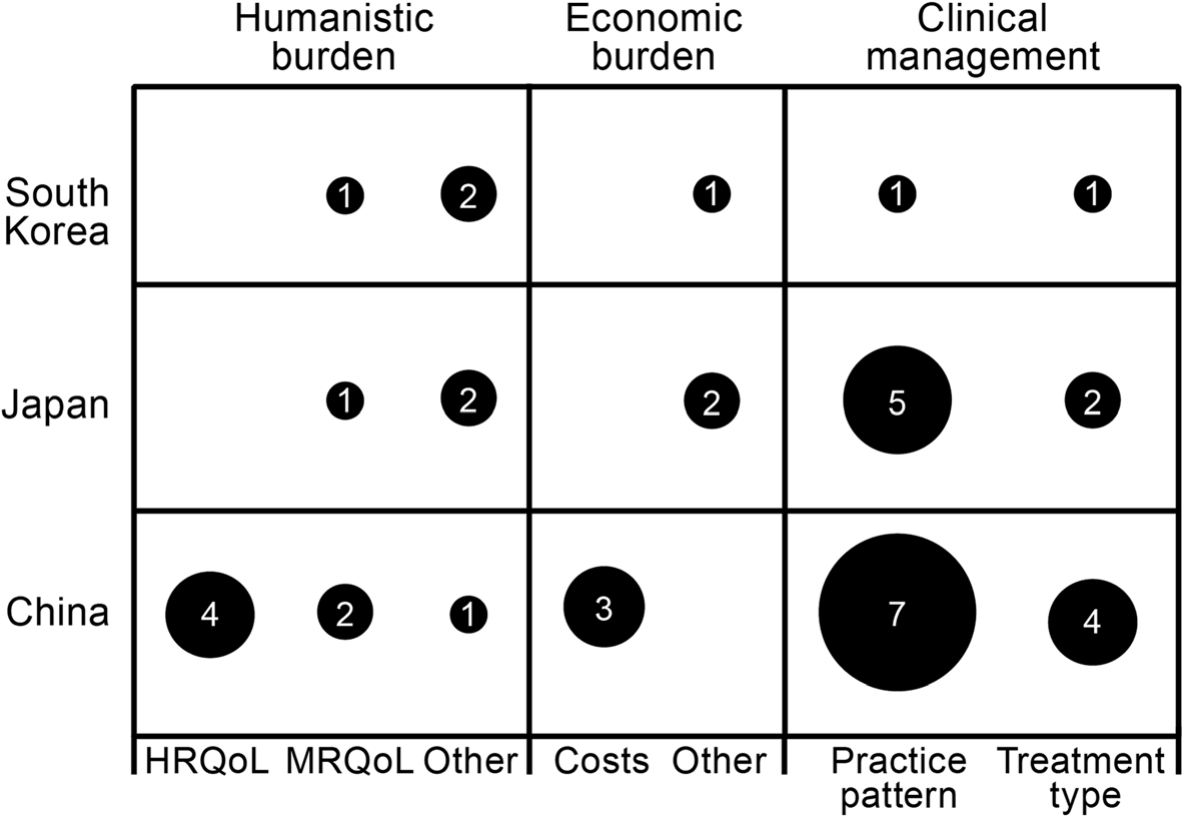
Number of studies reporting humanistic burden, economic burden, and clinical management of migraine. The size of the circle and numeral denotes the number of publications reporting each outcome; publications could be counted more than once. For burden, health-related quality of life (HRQoL) includes general health instruments, migraine-related quality of life (MRQoL) includes the Migraine Disability Assessment Questionnaire and Headache Impact Test-6, and Other includes various measures including bedrest, aspects of daily living, and school absence. Studies reporting prevalence only are not reported here

There was limited information on the burden of migraine in East Asia (Fig. 3). A total of 9 studies (11 publications) reporting the humanistic burden of migraine in adults were retrieved (Table 1): 5 population-based studies and 4 cross-sectional cohort studies of patients at headache clinics. The instruments used to assess humanistic burden included HRQoL instruments such as the EuroQoL-5 Dimensions (EQ-5D) questionnaire, the 36-item Short-Form health survey (SF-36), and the World Health Organization QoL-8 questionnaire (WHOQoL-8) [27, 29, 42, 46]. Migraine-related changes in QoL were assessed using country-specific versions of the Migraine Disability Assessment Questionnaire [MIDAS], the Migraine-Specific QoL [MSQ] Questionnaire, and the Headache Impact Test [HIT-6] [34, 46, 49, 55–57]. In addition, several studies reported on other non-specified instruments to assess effects of migraine on aspects of daily living [51, 55–57, 59].

**Table 1:** Studies reporting humanistic burden of migraine

Citation	Country/ region	Study design Study dates Migraine criteria	Migraine criteria	Migraine (n) Age, y % F	Main findings for participants with migraine
Yu, 2012 [29]	China / Mainland	Population-based 2009	ICHD-II	469 Mean 46.2 y 67.6 F	World Health Organization QoL-8 (migraine [*n* = 464] vs no headache; *P* < 0.05 for all comparisons): • Total score 25.7 vs 27.9 • Life quality 3.2 vs 3.4 • Health level 3.0 vs 3.6 • Daily life ability 3.4 vs 3.7 • Satisfied with yourself 3.5 vs 3.7 • Interpersonal relationships 3.8 vs 3.9 • Habitation condition 3.3 vs 3.5 • Daily life energy 3.0 vs 3.4 • Payment ability 2.6 vs 2.8
Wang, 2016 [27]	China / Mainland	Population-based 2013	ID Migraine Screener – Chinese version	102 Mean 51.5 y 84.3 F	HRQoL (SF-36) was significantly worse for respondents with migraine than those without. Domains significantly different (linear regression, *P* < 0.05) were: • Role physical −25.8 mean difference • Role emotional − 17.1 • General health − 13.0 • Bodily pain − 10.9 • Physical functioning − 3.8
Hung, 2006 [34]	China / Taiwan	Cross-sectional-other: headache clinic 2003–2004	ICHD-II	281 adults Mean 35.3 y 20–50 y 77.6 F	MIDAS-T Mean score: 34.2 ± 45.9 (severe disability) • Days missed from work/school 4.6 ± 9.9 • Reduced effectiveness days at work/school 8.2 ± 12.2 • Days missed from housework 7.1 ± 14.0 • Reduced effectiveness in housework 8.0 ± 12.1 • Days missed from family, social, or leisure activities 6.5 ± 13.9
Wang, 2013 [46]	China / Taiwan	Cross-sectional other: headache clinics 2011	Neurologist diagnosis / ICHD-II	331 adults Mean, 41 77.7 F	MIDAS scores for chronic migraine vs episodic migraine: • 46.1 ± 49.2 (grade IV–B) vs EM: 14.4 ± 23.4 (grade III), *P* < 0.001% MIDAS with severe disability: 59.3% vs 21.9% • % MIDAS with very severe disability: 41.3% vs 7.9% MSQ for chronic migraine vs episodic migraine: • Role function restrictive (56.4 ± 17.3 vs 70.8 ± 13.8, *P* < 0.001) • Role function preventive (70.0 ± 18.2 vs 81.4 ± 16.2, *P* < 0.001) • Emotional function scores (62.0 ± 23.0 vs 78.1 ± 16.8, *P* < 0.001) EQ-5D VAS chronic migraine vs episodic migraine: • 67.4 ± 18.7 vs 82.3 ± NR, *P* < 0.001
Wang, 2001 [42]	China / Taiwan	Cross-sectional other: headache clinic 1998–1999	IHS	193 adults Mean 41.8 y 80 F	Compared with SF-36 normative data (0–100) for Taiwanese women, migraine had the greatest effect on bodily pain and role emotional: • Role physical 77.6 vs 56.0 • Bodily pain 79.4 vs 49.7 • General health 63.3 vs 49.5 • Vitality 65.3 vs 50.9 • Social functioning 85.3 vs 67.9 • Role emotional 79.9 vs 54.2 • Mental health 71.8 vs 61.6
Sakai, 1997 [51]	Japan	Population-based NR	IHS	338 adults ≥15 y 79.0 F	74.2% had significant impairment in daily living (not defined): • Disability in social activity: severe (4.5%), moderate (27.5%), mild/none (68.0%) • Daily activity impairment: required bedrest always (4%), frequently required bedrest with severely impaired daily activity (30%), moderate impairment of daily activity (40%), minor impairment (21%), no impairment (5%)
Iigaya, 2003 [49]	Japan	Cross-sectional other: headache clinics 2000	IHS	99 patients with migraine and or TTH (72% had at least migraine) Mean 42.7 y 80.8 F	46.5% of patients were MIDAS grade I or II (minimal, mild, or infrequent disability), 22.2% were MIDAS grade III (moderate disability), and 31.3% were MIDAS grade IV (severe disability)
Roh, 1998 [59]	South Korea	Population-based 1996	IHS	272 adults ≥15 y 24.3 F	19.1% discontinued daily activities because of migraine 34.4% canceled work or social activities because of migraine
Kim, 2012; Kim, 2013; Chu, 2013 [55–57]	South Korea	Population-based 2009	ICHD-II	92 adults ≥19 y NR	Mean HIT-6 scores: 51.9 for women and 51.8 for men • Little or no impact, 42.8%–42.9% • Some impact, 25.3%–25.7% • Substantial impact, 13.0%–13.2% • Severe impact, 18.5%–18.7% Over the past 3 mo, patients with migraine experienced: • Restriction in activities for a mean of 2.7 days • Missing activities for a mean of 2.8 days
Adolescents/children					
Lu, 2000 [36]	China / Taiwan	Population-based 1998–1999	IHS	277 adolescents 13–15 y 58.8 F	30.4% of children with migraine were absent from school because of headache in the previous semester: 1–3 days 27%, ≥4 days 3.4%
Goto, 2017 [48]	Japan	Population-based 2012	IHS	131 adolescents 6–12 y: 42.5 F 12–15 y: 67.2 F	Feeling fed up or irritated, having difficulty concentrating were significantly more common (*P* = 0.010 and *P* = 0.017, respectively) in migraine than TTH For children with migraine, the number of days for the past 3 mo that disability affected school life, including school absences, arriving late, leaving early, or having difficulty participating in physical activities, ranged from 1.7 (SD 1.2) days to 3.8 (SD 3.7) days

*EQ-5D* European Quality of Life 5–Dimensions questionnaire; *F* female; *HIT-6* Headache Impact Test; *HRQoL* health-related quality of life; *ICHD-I/II/IIIβ* International Classification of Headache Disorders; *IHS* International Headache Society; *MSQ* Migraine-Specific Quality-of-Life Questionnaire; *MIDAS* Migraine Disability Assessment Questionnaire; *NR* not reported; *SD* standard deviation; *SF-36* Short-Form 36-item survey; *TTH* tension-type headache; *VAS* visual analog scale

When reported, the humanistic burden of migraine was substantial, suggesting that there are unmet needs for improved detection and treatment of migraine across East Asia (Table 1). Regression analysis of patients with chronic and episodic migraine from Taiwan showed chronic migraine to be significantly associated with higher levels of disability (MIDAS), lower MSQ scores (role function-restrictive, role function-preventive, and emotional function), and lower EQ-5D scores [46] than for episodic migraine. In addition, assessments of HRQoL conducted in China showed that migraine had negative effects on almost all SF-36 domains [27, 42] and that the SF-36 domains most significantly (linear regression, *p* < 0.05) affected were role-physical, role-emotional, bodily pain, physical functioning, and general health domains [27]. In addition, WHOQoL-8 total and domain scores were significantly (*t* test, *p* < 0.05) lower for patients with migraine compared with those without headache [29]. The domains most negatively affected were health level, daily life ability, and daily life energy.

In general, migraine was associated with moderate to severe levels of disability in approximately one-third of individuals from population-based studies in Japan and South Korea [51, 55–57, 59] (Table 1). In addition, mean MIDAS scores from studies conducted in China and Japan [34, 46, 49] indicated severe levels of disability among many patients attending headache clinics for migraine.

### Economic burden of migraine in adults

Very few studies on economic burden were retrieved (Table 2). Of the 6 studies retrieved, 3 provided an estimate of the direct or indirect medical costs of migraine or of costs associated with migraine work-related disability [29, 33, 38], with all suggesting that the economic impact of migraine was substantial at the time the studies were conducted.

**Table 2:** Studies reporting economic burden of migraine

Citation	Country/ region	Study design Study dates	Migraine criteria	Migraine (n) Age, y % F	Main findings
Yu, 2012 [29]	China / Mainland	Population-based 2009	ICHD-II	469 respondents Mean 46.2 y 67.6 F	Over the past 3 mo, average (SD): • Days of missed work 2.7 (7.5) • Impaired work days 4.0 (8.1) • Missed housework days 3.3 (7.1) • Impaired housework days 4.7 (8.6) Indirect costs due to migraine-related lost productivity: CNY 273.7 billion (USD 39.4 billion) Direct costs (diagnosis / treatment out-of-pocket expenses) per person affected year: CNY 729 Direct costs per year CNY 58.0 billion (USD 8.4 billion) Total annual cost of migraine per year: CNY 331.7 billion (USD 47.8 billion)
Fuh, 2008 [33]	China / Taiwan	Population-based 1997–1998	IHS migraine and modified migraine (IHS and attacks 2- to 4-h duration)	1813 employees NR 43.2 F 1809 non-employees NR NR	Over the past 1 y: • Median no. of missed days/employee, 2 days • Estimated median cost due to missed workdays/person in subjects with migraine vs without migraine: NTD 1667 vs NTD 0, *P* < 0.001 Projected annual number of missed workdays and economic loss attributed to migraine in 2005: • 3.7 million missed workdays/y (3.06 million in women, 0.64 million in men) • Estimated annual cost of migraine was NTD 4873/person • Estimated cost of NTD 4.6 billion/y due to lost workdays • Women accounted for ~ 80% of total cost (~ 56% of total cost attributed to women aged 35–54 y)
Tang, 2013 [38]	China / Taiwan	Cross-sectional other: retrospective case-control analysis of NHIRD 1996–2009	Refractory migraine (ICD-9-CM)	936 patients with refractory migraine vs 3743 non-migraine controls Mean 42 y 76 F 673 patients with refractory migraine vs 2202 with other migraine Mean age 41–42 y 79–80 F	All analyses adjusted for age, gender, urbanization level, income, and comorbidities Refractory migraine vs non-migraine adjusted for sociodemographic factors/comorbidity, mean (SD) Frequency of care: • Outpatient visits: 35.5 (33.2) vs 16.5 (14.4), *P* < 0.0001 • Emergency visits: 1.2 (1.3) vs 0.3 (0.3), *P* < 0.0001 • Hospital admission: 0.7 (0.8) vs 0.3 (0.3), *P* < 0.0001 • Hospital days: 7.1 (11.5) vs 2.4 (4.7), *P* < 0.0001 Annual total drug costs per person: NTD 19,752 (USD 608) vs NTD 8660 (USD 266), *P* < 0.0001 Annual total medical costs per person: NTD 57,932 (USD 1783) vs NTD 26,817 (USD 825), *P* < 0.0001 Refractory migraine vs other migraine adjusted for sociodemographic factors/comorbidity, mean (SD) Frequency of care: • Outpatient visits: 36.3 (23.0) vs 26.2 (12.3), *P* < 0.0001 • Emergency visits: 1.4 (1.6) vs 0.5 (0.5), *P* < 0.0001 • Hospital admission: 0.6 (0.8) vs 0.3 (0.3), *P* < 0.0001 • Hospital days 7.0 (10.9) vs 2.7 (5.1), *P* < 0.0001 Annual total drug costs per person: NTD 17,623 (USD 542) vs NTD 10,088 (USD 310), *P* < 0.0001 Annual total medical costs per person: NTD 54,678 (USD 1682) vs NTD 38,397 (USD 1181), *P* < 0.0001
Takeshima, 2004 [53]	Japan	Population-based 1999	IHS	342 adults ≥20 y 81.6 F	Over the past 3 mo: • 20.3% had time off work due to headache • 25.8% with MWA had time off work (mean no. of days: 1.8) • 19.5% with MOA had time off work (mean no. of days: 3.8) • 27.3% with MWA were unable to do housework (mean no. of days: 2.0) • 28.0% with MOA were unable to do housework (mean no. of days: 2.8)
Suzuki, 2014 [52]	Japan	Community-based 2007–2008	ICHD-II	704 Tokyo employees ≥20 y 77.4 F	25.1% had to miss work because of headache 2.7% could not work once per mo
Choi, 2018 [54]	South Korea	Cross-sectional other: prospective disease registry 2016–2018	ICHD-IIIβ	38^a^ adults Mean, 37.6 y 15.7 F	Employed patients with migraine compared with employed control patients without headache: • Experienced difficulty at work (63.9% vs 36.5%) • Had low productivity (33.3% vs 11.5%) • Greater sick leave (13.9% vs 3.8%) Migraine or TTH was significantly associated with difficulties at work after adjustment for health, anxiety, and stress (OR 3.05; 95% CI, 1.10–8.49; *P* = 0.032)

^a^ This study enrolled 143 patients with cluster headache, 38 age- and sex-matched patients with migraine or TTH, which included 5 patients had chronic migraine, 25 with episodic migraine, and 8 with TTH (4 chronic and 4 episodic), and 52 individuals without headache. Patients with cluster headache are not reported here

*CI* confidence interval; *CNY* Chinese yen; *F* female; *ICHD-I/II/IIIβ* International Classification of Headache Disorders; *IHS* International Headache Society; *MOA* migraine without aura; *MWA* migraine with aura; *NHIRD* Taiwan National National Health Insurance Database; *NR* not reported; *NTD* New Taiwanese dollar; *OR* odds ratio; *SD* standard deviation; *TTH* tension-type headache; *USD* United States dollar

From the population-based study conducted in mainland China in 2009, direct costs for out-of-pocket expenses for diagnosis and treatment per person affected year was estimated to be Chinese yuan renminbi (CNY) 729, resulting in direct costs per year of CNY 58.0 billion (United States dollars [USD] 8.4 billion). Individuals with migraine reported an average of 3 missed work or housework days and an average of 4 impaired work days and 9 impaired housework days over a 3-month period, resulting in an estimated CNY 273.7 billion (USD 39.4 billion) in indirect costs arising from migraine-related lost productivity. Combined, the total annual cost of migraine per year was estimated to be CNY 331.7 billion (USD 47.8 billion) [29].

Findings from a retrospective case-control analysis from Taiwan compared total drug and medical costs of patients with refractory migraine and with other migraine, and patients with refractory migraine and without migraine [38]. Health care utilization (outpatient visits, emergency visits, hospital admission, and length of stay) was significantly greater in patients with refractory migraine compared with other migraine types. The mean total annual medical costs per person with refractory migraine and other migraine were estimated at New Taiwan Dollars (NTD) 54,678 (USD 1682) and NTD 38,397 (USD 1181), respectively. Health care utilization was also significantly greater in patients with refractory migraine compared with patients without migraine. The mean total medical costs per person were NTD 26,817 (USD 825) and NTD 57,932 (USD 1783), respectively.

Findings from a population-based study conducted in Taiwan in 1997–1998 estimated that migraine was responsible for an annual cost of NTD 4873 (USD 149) per person and a total annual cost of NTD 4.6 billion (USD 140 million) per year due to lost work days. Women of all ages accounted for approximately 80% of costs, with women aged 35 to 54 years accounting for approximately 56% of costs [33].

The remaining studies, which were conducted between 1999 and 2018, reported observational outcomes related to missed work days and productivity from Japan [52, 53] and South Korea [54]. Overall, between 20% and 28% of individuals missed work or could not do housework because of headache, and more individuals with headache took greater sick leave and had lower productivity compared with individuals without headache (Table 2).

### Clinical management of migraine in adults

A total of 12 studies (14 publications) reporting information on clinical management of migraine in adults were retrieved (Table 3); 6 studies were population-based and 6 were other cross-sectional studies of headache patients or neurologists. Findings from the population-based studies reflected similar findings from the cross-sectional studies and, collectively, these studies showed a high degree of underdiagnosis and/or undertreatment (Table 3).

**Table 3:** Studies reporting clinical management of migraine

Citation	Country/region	Study design Study dates	Migraine criteria	Population (n)	Main findings for participants with migraine
Wang, 2011 and Li, 2012 [22, 28]	China / Mainland	Cross-sectional other: neurological outpatient department 2010	ICHD-II	401 patients with migraine	Practice pattern over the past 1 y: • 68.6% of patients had consulted a physician, 13.5% were diagnosed with migraine, 37.2% had not received any diagnosis Treatment over the past 3 mo: • 43.1% had not used analgesics for migraine, 11.7% were using analgesics ≥3 days/wk., none had used triptans, 2.7% had used preventive drugs
Liu, 2013 [24]	China / Mainland	Population-based 2009	ICHD-II	452 adults with migraine	Practice pattern over the past 1 y: • 52.9% of adults had consulted a physician for headache • 52.7% of adults who had a consultation for headache were undiagnosed • 13.8% were diagnosed with migraine, the remaining were diagnosed with other headache disorders Significant predictors of consultation for migraine were mild, moderate, or severe disability (HALT index) vs minimal HALT (0–5 days lost/3 mo): • Mild 6–10 days lost: adjusted OR 3.4 (95% CI, 1.6–7.4) • Moderate 11–20 days lost: adjusted OR 2.5 (95% CI, 1.2–5.4) • Severe > 20 days lost: adjusted OR 3.9 (95% CI, 1.9–8.1)
Lu, 2001 [35]	China / Taiwan	Population-based 1997–1998	> 15 headache days/mo for > 1 mo; > 4-h duration	108 adults with chronic daily headache	Practice pattern and treatment over the past 1 y: • 57% had consulted a physician for their headache • 41% consulted their family physician, 28% neurologist • 5% were treated with preventive drugs
Wang, 2000; Wang, 2001 [43, 44]	China / Taiwan	Population-based 1997–1998	IHS migraine and modified migraine (IHS + attacks of 2- to 4-h duration)	328 adults with migraine	Practice pattern over the past 1 y: • 54% had consulted a physician for the headache • 18% of these had been diagnosed with migraine by their physician Treating physicians • 29% general practitioners • 17% internists, 14% ENT specialists, 12% neurologists • 4.9% gynecologists, 4.6% ophthalmologists, 1.2% allergists, 2.7% other
Lu, 2006 [37]	China / Taiwan	Cross-sectional other: neurologists in Taiwan NR	NA	123 neurologists in Taiwan	31.7% of patients seen were outpatients with migraine Attitudes: • 88.5% reported headache to be an important part of their practice • 40.2% thought headache patients to be time-consuming • 86.9% reported patient satisfaction as an important consideration for treatment • 89.9% thought behavioral therapy to be an important part of treatment Treatment: • 69.9% agreed that preventive medication was indicated for ≥2 migraine attacks/wk., but 12.2% prescribed preventives for patients with ≥14 headaches/mo • Most commonly prescribed drugs were beta-blockers (96.7%), flunarizine (87.0%), tricyclic antidepressants (80.5%), and valproic acid (54.5%) • 32.5% had never prescribed triptans, mostly because of cost (35%)
Wang, 2008 [39]	China / Taiwan	Cross-sectional other: neurological clinics 2005	ICHD-II (MOA, MWA, probable)	755 patients with headache attending a neurology clinic for the first time	60% were diagnosed by neurologists with migraine 48% had MWA or MOA (ICHD-II) 71% had any migraine type (ICHD-II) of these, 23% were not diagnosed by neurologists as having migraine 57.4% diagnosed with any migraine (ICHD-II) had never been diagnosed with migraine previously
Wang, 2013 [46]	China / Taiwan	Cross-sectional other: headache clinics 2011	Neurologist diagnosis / ICHD-II	331 adults with migraine at neurology clinics	Over the past 3 mo for chronic migraine vs episodic migraine Health care professional evaluation of headache: 85.6% vs 81.7% • General practitioner: 34.3% vs 24.6% • Neurologist/specialist: 79.0% vs 80.6% • Emergency room visits: 21.0% vs 5.5% • Hospital admission: 4.8% vs 0% • Preventive medication: 48.5% vs 31.7%
Sakai, 1997 [51]	Japan	Population-based NR	IHS migraine and modified migraine (IHS + attacks of 2- to 4-h duration)	338 adults with IHS-defined or other defined migraine	69.4% had never consulted a physician for headache 11.6% were aware their headache was migraine 56.8% were taking OTC drugs 5.4% were taking prescription drugs 18.6% were taking OTC and prescription drugs 19.2% were not taking any medication
Takeshima, 2004 [53]	Japan	Population-based 1999	IHS	Adults 41 with MWA 301 with MOA	MWA vs MOA Most never consulted a physician for migraine 61.0% vs 71.8% Few continuously consulted a physician for migraine 7.3% vs 5.3% Main reasons for not consulting or not continuing to consult a physician: • Headache not severe enough 35.7% vs 29.3%; 38.5% vs 30.4% • Will improve spontaneously after standing 57.1% vs 56.9%; 30.8% vs 27.5% • OTC medication effective 21.4% vs 53.7%; 23.1% vs 30.4%
Kotani, 2004 [50]	Japan	Cross-sectional: other NR	IHS	35 patients with migraine at a general health clinic	Main reasons for not previously seeking medical attention: • 28.6% can endure symptoms without medication • 28.6% OTC medication is effective • 28.6% could not miss work • 25.7% could sleep and wake pain-free
Suzuki, 2014 [52]	Japan	Community-based 2007–2008	ICHD-II	704 employees in Tokyo	1.3% regularly visited their physicians 59.4% had never consulted with a physician about their headaches The most common reasons (*n* = 173) for stopping visits to a physician were: told their condition was not fatal (45.1%), unable to get adequate advice from their physician (20.2%), and no time (14.5%)
Roh, 1998 [59]	South Korea	Population-based 1996	IHS	272 adults with migraine	64.3% used medication for their migraine 92.8% used OTC medication 24.4% had consulted a physician for headache
Children/adolescents					
Lu, 2000 [36]	China/ Taiwan	Population-based 1998–1999	IHS	Children 13–15 y, 277	72.1% of children used painkillers for their headache 11.5% used painkillers ≥1 d/wk
Goto, 2017 [48]	Japan	Population-based 2012	ICHD-IIIβ (unilateral aura not included)	Children 6–12 y, 48 12–15 y, 37	Elementary school and junior high school students who reported disability due to migraine: • 44.9% and 47.9% had not had a medical consultation for their migraine • 30.6% and 8.3% had not received prescription medication for their migraine

*CI* confidence interval; *F* female; *HALT* Headache-Attributed Lost Time Index; *ICHD-I/II/IIIβ* International Classification of Headache Disorders; *ICD-9-CM* International Classification of Diseases, 9th revision, Clinical Modification; *IHS* International Headache Society; *NA* not applicable; *NR* not reported; *MOA* migraine without aura; *MWA* migraine with aura; *OTC* over-the-counter; *OR* odds ratio; *SD* standard deviation

#### China

Of the individuals or patients with migraine from China, 52.9% to 68.6% had consulted a physician previously, 37.2% to 52.7% who were diagnosed with headache had not been diagnosed with migraine previously, and 13.5% to 18% had been diagnosed with migraine previously (Table 3). In Taiwan, most patients with migraine from the general population consulted general practitioners (29% to 41%) and neurologists (12% to 28%) [35, 44]. For patients with refractory migraine at neurology clinics, most patients with chronic or episodic migraine (79.0% and 80.6%) had consulted a neurologist or specialist in the previous 3 months for their migraine, and 34.3% and 24.6% had consulted a general practitioner [46].

With regard to treatment, findings from a cross-sectional study of 401 migraine outpatients from a neurological clinic in mainland China showed that within the previous 3 months of being surveyed, 43.1% of individuals had not used analgesics, 2.7% had used preventives, and none had used triptans [22, 28]. In contrast, findings from an analysis conducted at a similar time of 311 migraine outpatients from Taiwan showed that 31.7% and 48.5% of patients with episodic and chronic migraine, respectively, had taken preventives [46].

One nationwide survey assessed the attitudes and migraine practice patterns of neurologists in Taiwan [37]. Of the 123 respondents to the survey, 88.5% indicated that headache was an important part of their practice, 40.2% found headache patients to be time-consuming, and 89.9% considered behavioral therapy to be an important part of treatment. With regard to practice patterns, 69.9% of the responding neurologists agreed that preventive medication was indicated for patients with ≥2 migraine attacks per week. In addition, approximately one-third (32.5%) had never prescribed triptans, mostly because of cost (35%) at the time this study was conducted. Almost all neurologists (97.4%) had encountered patients with headache who chronically used liquid forms of over-the-counter (OTC) medication with a combination of caffeine, acetaminophen, and other components.

#### Japan

Of individuals or patients with migraine from Japan [50–53], 59.4% to 71.8% had never consulted a physician previously, 1.3% to 7.3% regularly consulted a physician for their headache, and only 11.6% of individuals with migraine were aware that they had migraine. The main reasons for not consulting (or not consulting continuously) a physician for headache were that the headache was not severe enough or respondents could endure their symptoms, the headache improved spontaneously, OTC medication was effective, or the patient could not miss work [50, 53]. The most common reasons for no longer consulting a physician were being told their condition was not fatal, unable to get adequate advice, and no time [52].

With regard to treatment, 19.2% of patients from a population-based study were not taking any medication, 56.8% were taking OTC medication, 18.6% were taking OTC and prescription medication, and 5.4% were taking prescription medication only [51].

#### South Korea

There was limited information on health care utilization or clinical/treatment patterns from South Korea. Findings from one population-based study showed that only 24.4% of individuals with migraine had consulted a physician for headache, 64.3% were taking medication for their headache and, of these, most (92.8%) were using OTC medication [59].

### Burden and clinical management of migraine in children

For children, 2 population-based studies reporting information on burden or clinical management were retrieved: 1 from Taiwan [36] and 1 from Japan [48]. In both studies, migraine was associated with absenteeism from school, and difficulties in concentrating and in participating in physical activities [36, 48].

Findings from the study in Japan [48] showed that the numbers of school days over 3 months that were disrupted because of migraine among elementary and junior high school students, respectively, were 3.8 and 1.7 days for leaving early or arriving late, 2.7 and 2.6 days for absences, and 3.3 and 3.6 days for difficulty performing activities. Students reported feeling fed up, irritated, and had difficulty concentrating. Of the children who reported migraine-related disability, many (47.9% of 6- to 12-year-olds and 44.9% of 12- to 15-year-olds) had not consulted a physician for their migraine. In addition, 8.3% of junior high school students and 30.6% of elementary school students were not taking medication or only took OTC medication for their migraine.

In Taiwan [36], over the semester assessed, up to 30.4% of children with migraine had been absent from school because of headache; 27.0% were absent for 1 to 3 days, and 3.4% were absent for 4 or more days. Absence from school because of headache was significantly higher among students with migraine compared with those without migraine (30.4% vs 14.0%, *P* < 0.0001). A large proportion (72.1%) of children (13- to 15-year-olds) took painkillers for their migraine, with 11.5% taking painkillers more than once per week.

## Discussion

The findings from this comprehensive review of the literature suggest that there are unmet needs for migraine in terms of sufficient and appropriate diagnosis, and better management and therapies for treatment of migraine in East Asia. However, despite the number of publications retrieved from the literature review, there were significant gaps in the literature. The focus of most studies was the prevalence of migraine, with very little information on migraine burden or clinical management of migraine, particularly in South Korea or in children and adolescents. Although there were a considerable number of population-based studies, many were conducted before 2004, before the release of the ICHD-II criteria, and most were conducted more than 5 years ago. Only 3 studies reported on indirect productivity costs associated with migraine disability or direct costs associated with health care utilization or medical costs [29, 33, 38]. Each of these studies were from China and reported on information collected at least 10 years ago.

In 2016, the worldwide, age-standardized prevalence of migraine was estimated to be 14.4%: 18.9% for women and 9.8% for men [6]. Findings from the population-based studies retrieved in our literature review showed that migraine prevalence among adults in East Asia ranged from 6.0% to 14.3% in non-elderly adults, which is consistent with worldwide estimates of migraine prevalence [6] and a previous study conducted among Asia-Pacific countries up until 2013 [16]. Consistent with worldwide studies on migraine disability [6], the peak prevalence of migraine in East Asia was among adult women aged 30 to 49 years. In addition, findings from the current study showed that the prevalence of migraine in children increased with age to approximately 8.5% at 15 years. An analysis of 64 population-based studies worldwide suggests that the prevalence of migraine in children is 9.1% [14], which is higher than reported for children in this study. However, this analysis did not account for the many different age groups of the populations assessed or the different criteria used to identify migraine across studies. Given that the populations of China, Japan, and South Korea collectively represent 21% of the global population, these findings highlight the substantial number of people affected by migraine in East Asia.

A major finding from this study is that there appeared to be a low level of disease awareness and use of prescription medication for migraine in East Asia at the time the studies were conducted. Many patients identified as having migraine had not consulted or were not currently seeing a physician for their migraine, and many patients had not been diagnosed with migraine previously, suggesting that migraine is underdiagnosed and, therefore, undertreated. Although the search was limited to publications from 1988 onward, after the availability of triptans for management of acute migraine pain, most patients were not taking medication or were taking OTC medication, and use of both triptans and preventives appeared to be low. These findings are generally consistent with more recent studies, published after this review was conducted, which have shown low prescribing rates for triptans in China [60] and low prescribing rates for preventives in China and Japan [60–62]. However, a recent study on real-world treatment patterns suggests that triptans are now commonly prescribed for patients with migraine in Japan [61].

Although use of complementary medicine and physical treatments such as acupuncture were outside the scope of this review, these therapies have a major role in the management of patients with migraine in East Asia [60, 63, 64]. In East Asia, complementary medicine may comprise traditional herbal preparations or patent medicines (typically prepared as high-quality extracts of herbal treatments). In Japan, traditional patent medicines are prescribed in combination with Western prescription medicine for management of various types of pain including headache and migraine [63] and a recent analysis of the China Health Insurance Association (CHIRA) medical insurance claims database has shown that approximately 25% of patients are prescribed Chinese patent medicine for migraine [60].

The impact of inadequate treatment on migraine burden and health care utilization was assessed in a global survey of 11,266 individuals with migraine experiencing ≥4 migraine headaches per month, in which ≥80% of respondents had failed preventive treatment at least once [7]. In this survey, 87% of individuals reported negative effects in their private, social, and professional lives, and high levels of health care utilization. Within a 6-month period, 58% had had a brain scan, and within a 12-month period, 38% had visited an emergency department and 23% had stayed in hospital overnight. In addition, findings from the International Burden of Migraine Study [1], which surveyed 8726 individuals from predominantly Caucasian populations with chronic or episodic migraine, showed that approximately 50% reported moderate or significant levels of disability (MIDAS grade III or IV) and significantly negative effects on HRQoL for those with chronic migraine. Consistent with studies in predominantly Caucasian populations [1, 65], the humanistic burden of migraine in East Asia from the available studies has suggested that there are unmet needs for improvements in diagnosis, management, and therapies for treatment of migraine across East Asia. When reported, mean MIDAS scores for headache clinic outpatients in China and Japan indicated severe levels of disability [34, 46, 49]. In addition, HRQoL (SF-36) domains most negatively affected were bodily pain, physical functioning, role physical, and role emotional, which is consistent with findings showing significant impairments in daily activities [27, 46]. For adults, this migraine-related disability translates to significant levels of absenteeism and reduced effectiveness in the workplace and at home, and for children, significant negative effects on school attendance and participation in physical activities.

Although evidence on the economic costs of migraine in this study was limited to 3 studies from China (Table 2), the findings demonstrated that migraine was associated with costs of billions of dollars per year arising from lost productivity, drug costs, and direct medical costs. However, in contrast to studies from the United States [66] and Europe [67], the greatest proportion of the costs of migraine in China were attributed to lost productivity [29]. In the United States, direct medical costs were estimated to account for at least 60% of the total costs of migraine [66], whereas in China, lost productivity was estimated to account for 82% of total costs [29]. The high costs arising from lost productivity have also been reported in Japan, where lost productivity related to migraine was reported to account for 288 billion yen per year [68].

The strengths of this comprehensive review are that it has provided a detailed overview of the current status of evidence in the peer-reviewed literature on migraine burden and clinical management in East Asia. However, despite the number of studies retrieved, the available evidence was limited, particularly for children. Comparisons across countries and regions need to take into account cultural factors in patient responses to disability, and the socioeconomic status of the populations assessed in each country. However, because of the wide variation in study types, populations, and analysis methods, it was not possible to compare findings between countries.

## Conclusion

In conclusion, despite the high prevalence and significant level of migraine-associated disability reported in population-based studies in East Asia, there is little or no information on economic burden associated with migraine or migraine disability, work-related productivity losses, or costs associated with treatment or undertreatment. Combined with the high prevalence of migraine across all countries included in this review, the significant levels of humanistic burden among the available studies suggest that there are substantial unmet needs for migraine with regard to appropriate diagnosis, and better management of and therapies for treatment of migraine across East Asia. However, more recent evidence is required to confirm current unmet needs in each country.

## Supplementary information

**Additional file 1.** Population-based studies reporting prevalence of migraine

## Data Availability

All data generated or analyzed during this study are included in this published article [and its supplementary information files].

## Abbreviations

CNY: Chinese yuan renminbi
EQ-5D: EuroQoL-5 Dimensions
HIS: International Headache Society
HRQoL: Health-related quality of life
ICHD: International Classification of Headache Disorders
MIDAS: Migraine Disability Assessment Questionnaire
MSQ: Migraine-Specific QoL Questionnaire
NTD: New Taiwan Dollars
OTC: Over-the-counter
SF-36: 36-item Short-Form health survey
USD: United States dollars
