# Supplementary material for: Prevalence, burden, and clinical management of migraine in China, Japan, and South Korea: a comprehensive review of the literature
Source: J Headache Pain. 2019 Dec 5;20(1):111. doi: 10.1186/s10194-019-1062-4 (PMC6896325; doi:10.1186/s10194-019-1062-4)
Supplement: Supplementary file 1 — Additional file 1. Population-based studies reporting prevalence of migraine [file 10194_2019_1062_MOESM1_ESM.pdf]

Supplementary Table 1. Population-based studies reporting prevalence of migraine.

Citation	Country/ region	Data collection Study dates	Migraine criteria	Population Total/analysed	Age (years) Gender (% F)	Prevalence
Elderly adults						
Wang, 1997 (43)	China / Taiwan	Door-to-door questionnaire 1993–1994	IHS	2003/1533 RR 77%	65+ y 76 F	Crude: 5.2%<br>1-y: 3.0% (95% CI, 2.1%–3.9%)<br>Peak: 65–69 y 4.1% (6.1% F, 1.9% M)
Zhang, 2016 (28)	China / Mainland	Door-to-door questionnaire 2014–2015	ICHD-IIIβ	5248/5038 RR 96%	60+ y NR	1-y: 0.9% (95% CI, 0.6–1.1)<br>Peak: 60–69 y 1.2%
Adults						
Yu 2012 (27)	China / Mainland	Door-to-door questionnaire 2009	ICHD-II	NR/5041 RR 94.1%	18–65 y 49.2 F	1-y: 9.3% (95% CI, 8.5%–10.1%)<br>Peak[a]: 40–49 y 12% (16% F, 8% M)
Huang, 2013 (18)	China / Mainland	Face-to-face questionnaire 2009	ICHD-II	6328/5519 RR 87.2%	≥15 y 56.9 F	Crude: 10.5%<br>Standardised: 9.6%<br>Peak: 40–49 y 11.3% (14.5% F, 6.0% M)
Luo, 2014 (23)	China / Mainland	Face-to-face questionnaire 2009	ICHD-II	570/554 RR 97.2%	Mean (2 sites): 40–42 y 42–47 F	1-y per region:<br>8.3% (95% CI, 6.0%–11.0%)<br>14.3% (95% CI, 9.0%–19.0%)<br>Peak: NR
Wang, 2016 (25)	China / Mainland	Door-to-door questionnaire 2013	ID-Migraine Screener – Chinese version	2588/1143 RR 44.2%	Mean 50.7 y 61.9 F	Crude: 8.9% (not ICHD-III confirmed)<br>Peak: 35–44 y 12%[a] (17.4% F, <5% M[a])
Lin, 2018 (21)	China / Mainland	Face-to-face questionnaire 2014–2015	ICHD-III	9134/7860 RR 86.1%	≥15 y 52.3 F	Crude: 9.1%<br>Peak: 40–49 y 11.0%
Wong, 1995 (30)	China / Hong Kong	Telephone questionnaire 1998	IHS	3156/1436 RR 45.5%	≥15 y 55.3 F	Crude: 4.7% (95% CI, 3.7%–6.0%)<br>Peak: NR
Wang, 2000 (41)	China / Taiwan	Face-to-face questionnaire 1997–1998	IHS migraine and modified migraine (IHS + attacks of 2-to 4-h duration)	4434/3377 RR 76.2%	≥15 y 46.6 F	Crude<br>IHS migraine, 7.7% (95% CI, 6.8%–8.6%)<br>Modified migraine, 2.0%<br>Crude, 9.7% (95% CI, 8.7%–10.7%)

Citation	Country/ region	Data collection Study dates	Migraine criteria	Population Total/analysed	Age (years) Gender (% F)	Prevalence
						Peak: 30–34 y 13.5% (35–39 y 16.0% F, 25–29 y 8.3% M)
Takeshima, 2004 (51)	Japan	Door-to-door questionnaire 1999	IHS	5758/4795 RR 83.4%	≥20 y 54.6 F	1-y: 6.0% (95% CI, 5.4%–6.6%) Peak MOA: 40–49 y 17.6% F; 30–39 y 2.8% M
Sakai, 1997 (49)	Japan	Telephone survey, postal questionnaire NR	IHS migraine and modified migraine (IHS + attacks of 2- to 4-h duration)	4029/1029 RR 64.4%	≥15 y 66.8 F	Crude IHS migraine 6.0% Crude Modified migraine 8.4% Peak[a]: 30–39 y 20% F, 20–29 y 7% M
Roh, 1998 (57)	South Korea	Telephone survey, postal questionnaire 1996	IHS (MOA, MWA, and migrainous disorder not fulfilling the criteria above)	2500/1701 RR 68.3% telephone; 804/380 RR 47.3% postal	≥15 y 50.7 F	Crude all codes: 22.3% MWA: 10.4%, MOA: 11.0% Peak: 15–19 y 34.7% F, 28.5% M
Kim, 2012 and Chu, 2013 (53,54)	South Korea	Face-to-face questionnaire 2009	ICHD-II	4054/1507 RR 37.2%	≥19 y NR	1-y: 6.1% Peak[a]: 40–49 y 7% (11% F, 4% M)
Young adults						
Gu, 2018 (17)	China / Mainland	Self-reported questionnaire 2016	ID-Migraine Screener – Chinese version	1020/986 RR 96.7%	Mean 22.4 y 63 F	1-y: 7.9% (95% CI, 6.2%–9.6%) Peak: NR
Wang, 2015 (24)	China / Mainland	Self-reported questionnaire 2012	ID-Migraine Screener – Chinese version	5129/4406 RR 85.9%	Mean 21.5 y 67.6 F	Crude 9.0% (95% CI, 8.2%–9.8%) Peak: NR
Children/adolescents						
Jin, 2013 (19)	China / Mainland	School-based questionnaire NR	ICHD-II-revised	4823/4812 RR 99.8%	7–15 y 46.9 F	Crude 4.3% Peak: NR
Kong, 2001 (29)	China / Hong Kong	School-based questionnaire 1999	IHS (migraine and migraine excluding ≥5 attacks)	2156/2120 RR 98.3%	5–16 y 47.4 F	Crude 1.2% (95% CI, 0.7–1.6) Peak: 12–13 y 5.5%

Citation	Country/ region	Data collection Study dates	Migraine criteria	Population Total/analysed	Age (years) Gender (% F)	Prevalence
Lu, 2000 (34)	China / Taiwan	School-based questionnaire 1998-1999	IHS (MOA excluding ≥5 attacks)	4436/4064<br>RR 91.6%	13–15 y<br>51.2 F	Crude:<br>13 y 4.8% (95% CI, 3.6%–6.0%)<br>14 y 7.1% (95% CI, 5.7%–8.5%)<br>Peak: 15 y 8.4% (95% CI, 7.0%–9.8%)
Wang, 2005 (38)	China / Taiwan	Questionnaire interviews 1999-2001	IHS (MOA excluding ≥5 attacks)	23,433/13,426<br>RR 97%–99%	13–15 y<br>47–48 F	1-y:<br>1999 5.2% (95% CI, 4.7%–5.7%)<br>2000 6.8% (95% CI, 6.3%–7.4%)<br>2001 7.4% (95% CI, 6.8%–8.0%)<br>Peak 2001: 15 y (Grade 9): 8.5% (11% F, 6.3% M)
Wang, 2009 (39)	China / Taiwan	School-based questionnaire 2005	ICHD-II (MOA, MWA, probable)	4259/3963<br>RR 93.0%	13–15 y<br>48.5 F	Crude<br>All 23.4%, MWA 3.5%, MOA 8.7%, Probable 11.2%<br>Peak: NR
Goto, 2017 (46)	Japan	School-based questionnaire 2012	ICHD-IIIβ (unilateral aura was not included)	3404/3285<br>RR 96.5%	6–12 y: 50.9 F<br>12–15 y: 47.5 F	Crude:<br>6–12 y: 3.5% (n=73)<br>12–15 y: 5.0% (n=58)<br>Peak: NR
Ando, 2007 (45)	Japan	School-based questionnaire 2004	ICHD-II for children	6869/6472<br>RR 94.2%	12–15 y<br>48.3 F	Crude: 4.8% (313/6470)<br>Peak: NR
Rho, 2012 (56)	South Korea	School-based questionnaire 2009	IHS for children	5360/5039<br>RR 94.0%	6–18 y<br>52.3 F	1-y prevalence 8.7%<br>6–12 y 4.9%<br>13–15 y 9.0% [a]<br>Peak: 16–18 y 14.2%

CI, confidence interval; F, female; ICHD-II/IIIβ, International Classification of Headache Disorders; IHS, International Headache Society; M, male; MOA, migraine without aura; MWA, migraine with aura; NR, not reported; OR, odds ratio; RR, response rate.

[a] Estimated from graph in publication.
